# Over-Expression of UV-Damage DNA Repair Genes and Ribonucleic Acid Persistence Contribute to the Resilience of Dried Biofilms of the Desert Cyanobacetrium *Chroococcidiopsis* Exposed to Mars-Like UV Flux and Long-Term Desiccation

**DOI:** 10.3389/fmicb.2019.02312

**Published:** 2019-10-11

**Authors:** Claudia Mosca, Lynn J. Rothschild, Alessandro Napoli, Fabrizio Ferré, Marco Pietrosanto, Claudia Fagliarone, Mickael Baqué, Elke Rabbow, Petra Rettberg, Daniela Billi

**Affiliations:** ^1^Department of Biology, University of Rome Tor Vergata, Rome, Italy; ^2^Earth Sciences Division, NASA Ames Research Center, Mountain View, CA, United States; ^3^Department of Pharmacy and Biotechnology, University of Bologna Alma Mater, Bologna, Italy; ^4^Astrobiological Laboratories Research Group, German Aerospace Center, Institute of Planetary Research, Management and Infrastructure, Berlin, Germany; ^5^German Aerospace Center, Institute of Aerospace Medicine, Cologne, Germany

**Keywords:** habitability and astrobiology, anhydrobiosis, desert cyanobacteria, Mars UV simulation, DNA repair

## Abstract

The survival limits of the desert cyanobacterium *Chroococcidiopsis* were challenged by rewetting dried biofilms and dried biofilms exposed to 1.5 × 10^3^ kJ/m^2^ of a Mars-like UV, after 7 years of air-dried storage. PCR-stop assays revealed the presence of DNA lesions in dried biofilms and an increased accumulation in dried-UV-irradiated biofilms. Different types and/or amounts of DNA lesions were highlighted by a different expression of *uvrA, uvrB, uvrC, phrA,* and *uvsE* genes in dried-rewetted biofilms and dried-UV-irradiated-rewetted biofilms, after rehydration for 30 and 60 min. The up-regulation in dried-rewetted biofilms of *uvsE* gene encoding an UV damage endonuclease, suggested that UV-damage DNA repair contributed to the repair of desiccation-induced damage. While the *phrA* gene encoding a photolyase was up-regulated only in dried-UV-irradiated-rewetted biofilms. Nucleotide excision repair genes were over-expressed in dried-rewetted biofilms and dried-UV-irradiated-rewetted biofilms, with *uvrC* gene showing the highest increase in dried-UV-irradiated-rewetted biofilms. Dried biofilms preserved intact mRNAs (at least of the investigated genes) and 16S ribosomal RNA that the persistence of the ribosome machinery and mRNAs might have played a key role in the early phase recovery. Results have implications for the search of extra-terrestrial life by contributing to the definition of habitability of astrobiologically relevant targets such as Mars or planets orbiting around other stars.

## Introduction

Our knowledge of the limit of life’s adaptability to extreme environments is mandatory for identifying habitable planets and moons in the Solar System and planetary systems orbiting around other stars (Schulze-Makuch et al., 2017). Dryness is one of the main factors threatening life since water removal causes membrane phase transition and production of reactive oxygen species that cause lipid peroxidation; this leads to protein oxidation and DNA damage, which are lethal to the majority of the organisms (França et al., 2007). Nevertheless, a few organisms called anhydrobiotes, survive desiccation by stabilizing their sub-cellular structures and entering a metabolic dormancy until water is available again (Crowe et al., 1992). As a by-product of desiccation tolerance, anhydrobiotes are also radiation tolerant, being able to cope with high doses of UV and ionizing radiation that are not present in nature (Cox and Battista, 2005).

Despite the interest in anhydrobiotes, it is not yet known how long they can persist in the air-dried state and which levels of radiation doses they can experience without dying. Knowing their desiccation endurance threshold is relevant to understanding not only the limits of life on Earth but also to assessing the potential habitability of astrobiologically relevant targets such as Mars and rocky exoplanets with transient availability of liquid water (Wilhelm et al., 2018). In addition, the identification of their UV resistance threshold has implications when tackling the surface habitability of rocky planets with elevated UV radiation fluxes (O’Malley-James and Kaltenegger, 2017).

Anhydrobiotic cyanobacteria of the genus *Chroococcidiopsis* possess a remarkable resistance to desiccation and radiation that has extended the limits of life, as we know it, in several new directions. *Chroococcidiopsis* sp. CCMEE 029 isolated from the Negev Desert survived 4 years of air-drying on the top of polycarbonate filters or spotted on Petri dishes (Billi, 2009; Fagliarone et al., 2017) and 13 years on desiccated agar (Cockell et al., 2017). When dried, *Chroococcidiopsis* sp. CCMEE 029 withstood up to 24 kGy of γ-radiation (Verseux et al., 2017), whereas dried monolayers could cope with 15 kJ/m^2^ of a Mars-like UV flux (Cockell et al., 2005). This resistance was further extended by the survival of dried biofilms exposed to 1.5 × 10^3^ kJ/m^2^ of a Mars-like UV flux (Baqué et al., 2013). The exposure of microbial biofilms to Mars-like conditions was carried out during ground-based simulations performed in the context of the Biofilm Organisms Surfing Space (BOSS) project. This project aimed to assess whether biofilms are better suited than planktonic counterparts to cope with space and Mars-like conditions by taking advantage of the exposure to low Earth orbit conditions outside the International Space Station (Rabbow et al., 2017). The survival of dried *Chroococcidiopsis* biofilms exposed to 1.5 × 10^3^ kJ/m^2^ of a Mars-like flux was previously ascribed to the shielding provided to the bottom-layer cells by the top-layer cells, killed by the UV radiation, and to abundant extracellular *exopolysaccharides* (Baqué et al., 2013).

However, several aspects of the desiccation and radiation tolerance of desert strains of *Chroococcidiopsis* remain poorly characterized. For instance, it is not known whether dried cells irradiated with high radiation doses can recover when rewetted after prolonged desiccation. The survival of dried biofilms of *Chroococcidiopsis* sp. CCMEE 029 exposed to 1.5 × 10^3^ kJ/m^2^ of a Mars-like UV flux was reported soon after the ground-based simulation (Baqué et al., 2013); while their survival after exposure to Mars-like simulations in low Earth orbit was assessed on 2.5-year-old samples, due to the EXPOSE-R2 space mission duration, e.g., about 900 days from launch to sample return to the lab (Billi et al., 2019a).

In the present work, the survival limits of *Chroococcidiopsis* sp. CCMEE 029 were challenged by rewetting dried biofilms and dried biofilms exposed to 1.5 × 10^3^ kJ/m^2^ of a Mars-like UV after 7 years of air-dried storage. The presence of DNA lesions was evaluated by means of polymerase chain reaction (PCR)-stop assay. Viability was tested by assessing the capability of entering cell division and by staining with a redox dye after rehydration. An *in silico* survey of the genome was performed to search for genes encoding proteins involved in photoreactivation, nucleotide excision repair, and UV damage endonuclease (UvsE)-dependent excision repair, that are known to be associated with UV-induced DNA damage repair (Goosen and Moolenaar, 2008). The role of the identified UV-damage repair genes in the early phase recovery of dried-UV-irradiated biofilms and dried biofilms was investigated by real-time quantitative polymerase chain reaction (RT-qPCR) performed after 30 and 60 min of rehydration.

## Materials and Methods

### Organism and Culture Conditions

*Chroococcidiopsis* sp. CCMEE 029 (hereafter *Chroococcidiopsis*) was isolated by Roseli Ocampo-Friedmann from cryptoendolithic growth in sandstone in the Negev Desert (Israel) and is now maintained at the University of Rome Tor Vergata, as part of the Culture Collection of Microorganisms from Extreme Environments (CCMEE) established by E. Imre Friedmann. *Chroococcidiopsis* sp. CCMEE 029 was reported to be not axenic (Billi et al., 1998), although routinely, colony transfers reduced the bacterial contamination to about 0.0001% (Billi et al., 2019b). Cultures were routinely grown in BG-11 medium (Rippka et al., 1979) at 25°C, under a photon flux density of 40 μmol/m^2^ s^1^ provided by fluorescent cool-white bulbs.

### Desiccation, Mars-Like UV Irradiation, and Rehydration

Biofilms were obtained by growing *Chroococcidiopsis* cells on top of BG-11 agarized medium in Petri dishes sealed with Parafilm. After 2 months of growth, the Parafilm was removed and biofilms were allowed to air-dry for about 15 days. Finally 12-mm-in-diameter disks were cut out of biofilms and shipped to the Planetary and Space Simulation facilities (PSI) at the Radiation Biology Department of the Institute of Aerospace Medicine/Microgravity User Support Center (DLR Cologne, Germany). Samples in triplicates were integrated in the DLR 16-well aluminum sample carriers and exposed to solar simulator SOL2000 with a fluence of 1,370 W/m^2^ in the 200- to 400-nm wavelength range (Rabbow et al., 2016). Dried biofilms were irradiated with a dose of 1.5 × 10^3^ kJ/m^2^ obtained in 18 min of exposure, and then kept in the dark at room temperature until sent back to Tor Vergata University for analysis. Part of these samples were previously analyzed in the context of the Biofilm Organisms Surfing Space experiment (Baqué et al., 2013), the remaining samples were stored in the laboratory sealed in plastic envelopes, in the dark and at room temperature.

**Figure 1:**
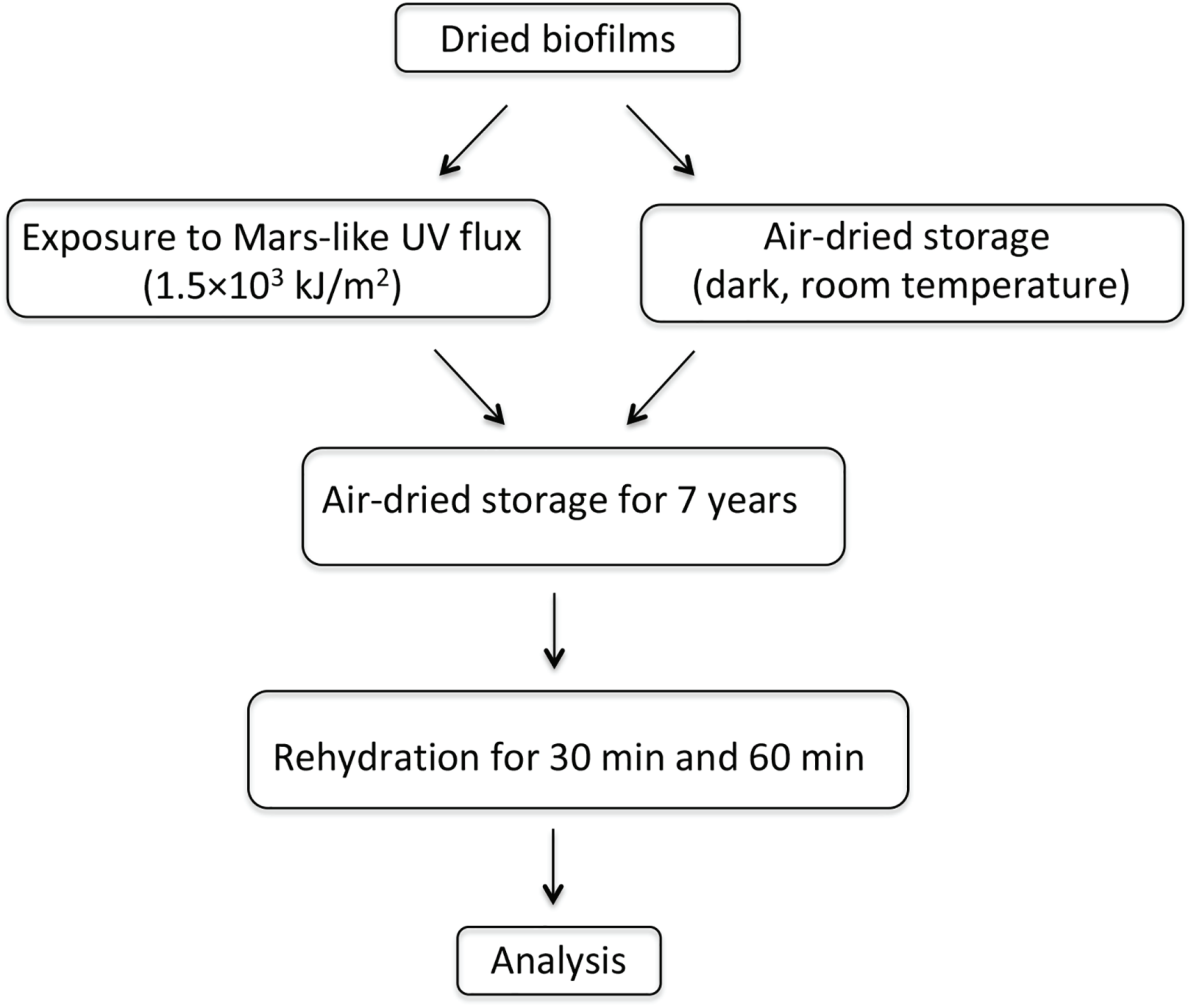
Experimental scheme.

After 7 years of air-dried storage, the following analyses were performed: (1) cell morphology of dried biofilms and dried-UV-irradiated biofilms was evaluated by confocal laser scanning microscopy (CLSM), using liquid cultures as control; (2) viability of dried biofilms and dried-UV-irradiated biofilms was tested by assessing their capability of entering cell division and by staining with a redox dye after rewetting; (3) gene expression was evaluated in dried-rewetted biofilms after 30 and 60 min of rehydration, by using dried biofilms (0-min recovery) as control; (4) gene expression was evaluated in dried-UV-irradiated biofilms after 30 and 60 min of rehydration, by using as control dried-rewetted biofilms at the same rehydration time; and (5) DNA damage was quantified in dried biofilms and dried-UV-irradiated biofilms by performing PCR-stop assays; liquid cultures were used a control. A schematic experimental plan is shown in Figure 1.

### Cell Morphology and Viability

Cells from dried biofilms and dried-UV-irradiated biofilms were observed with a CLSM (Olympus Fluoview 1,000 Confocal Laser Scanning System). Images were taken using a 60× objective and photosynthetic pigment autofluorescence was investigated by exciting the cells with a 543- and a 635-nm laser and collecting the emission from 645-nm, or from 553-nm, to 800-nm emission range.

Viability was assessed: (1) by inoculating biofilm fragments (about 25 mm^2^) into 2 ml of liquid BG-11 medium and measuring cell densities with a spectrophotometer after 3 months of growth under routine conditions and (2) by staining with 2-(4-Iodophenyl)-3-(4-nitrophenyl)-5-phenyl tetrazolium chloride (Sigma Aldrich, Saint Louis, MO, USA) after rehydration for 30 min, 60 min, and 72 h, as previously reported (Billi, 2009).

### Genomic DNA Extraction and Damage Evaluation by Polymerase Chain Reaction-Stop Assay

Genomic DNA was extracted by using a method developed to reduce bacterial contamination and based on lysozyme treatment, osmotic shock, and DNase I treatment, while *Chroococcidiopsis* lysis, due to its lysozyme resistance, is achieved by adding hot phenol and glass beads (Billi et al., 1998). Here lysozyme and DNase I steps were avoided because PCR-stop assays were performed by using *Chroococcidiopsis*-specific primers.

The extracted DNA was quantified by using the NanoDrop Lite Spectrophotometer (Thermo Fisher Scientific, Waltham, MA, USA) and 6 ng were used in 12-μl PCR reaction mixtures as follows:

#### Short-Fragment Polymerase Chain Reaction Amplification

A 1,027-bp fragment of the 16S rRNA gene was amplified by using 0.5 μM each (final concentration) of primers CYA-359F (5′-GGGGAATTTTCCGCAATGG-3′) and CRev (5′-ACGGGCGGTGTGTAC-3′), and 6 μl of MyTaq™ Red Mix (Bioline Meridian Life Science, Memphis, TN, USA). PCR conditions were as follows: 94°C for 3 min; 35 cycles of 94°C for 1 min, 45°C for 1 min, and 72°C for 3 min; and 7 min at 72°C.

#### Long-Fragment Polymerase Chain Reaction Amplification

A 4-kbp genome fragment was amplified using 0.5 μM each (final concentration) of primers Chroo-4 K-2-F (5′-GCTACTCGTTGCTTTGCGTC-3′) and Chroo-4 K-2-R (5′-TTCCCCATACTTTGCTTCCCA-3′), and 6 μl of High-Fidelity Master Mix (Thermo Fisher Scientific, Waltham, MA, USA). PCR conditions were as follows: 98°C for 3 min; 30 cycles of 98°C for 30 s, 65°C for 1 min, and 72°C for 2 min; and 7 min at 72°C.

Each one of the 12-μl PCR reaction mixtures was loaded onto 1.5% agarose gel containing 0.5 mg/ml ethidium bromide, subjected to electrophoresis for about 1 h at 90 V, and visualized with a trans-illuminator.

#### Real-Time Quantitative Polymerase Chain Reaction

A 1,027-bp fragment of the 16S rRNA was amplified in 25-μl reaction mixtures containing DNA template (5 ng), 12.5 μl of qPCR cocktail (iQ SYBR Green Supermix, Bio-Rad Laboratories, Hercules, CA, USA), and 0.5 μM (final concentration) of primers 16SF (5′-GGGGAATTTTCCGCAATGGGCG AAAGCCTGACGGAG-3′) and 16SR (5′-CGGGCGGTGTGTACAAGGCCCGGG AACGTATTCACC-3′). A real-time PCR detection system (iQ5, Bio-Rad Laboratories, Hercules, CA, USA) was programmed to operate as described (Baqué et al., 2013). PCR protocols were carried out performing *n* ≥ 3 replicates.

### Identification UV-Induced DNA Repair Genes

Genomic DNA extracted from liquid cultures as previously described (Billi et al., 1998) was sequenced by using Illumina Solexa technology (CD Genomics NY USA), obtaining around 1.7 M 300×2 paired-end reads. After quality control (by using FastQC, https://www.bioinformatics.babraham.ac.uk/projects/fastqc/), reads were trimmed and adapter sequences removed, by using Trimmomatic (Bolger et al., 2014) with parameters LEADING:20, TRAILING:20, AVGQUAL:28, MINLEN:25. Then, trimmed reads were checked for contaminants by using BLAST against the NCBI nt database, and discarding all reads having a significant match (coverage > 80%, e-value < 10^−4^) with species other than Cyanobacteria. Surviving reads were assembled using Velvet version 1.2.10, a *de novo* assembler for next-generation sequencing data that employ de Bruijn graphs, with the following parameters: K-mer length 181, expected coverage (exp_cov) 8, and coverage cutoff (cov_cutoff) 7. The obtained contig genomic sequences were annotated using Prokka (Seemann, 2014), a prokaryotic gene annotator, using the interface provided by the Galaxy-based framework Orione (Cuccuru et al., 2014),^1^ by setting the following parameters: similarity e-value cutoff 1e-06, minimum contig size 200, and using the pre-set for improving gene predictions for highly fragmented genomes.

### RNA Extraction and Real-Time Quantitative Polymerase Chain Reaction

Total RNA was extracted by using 1 ml of TRI Reagent (Sigma Aldrich, Saint Louis, MO, USA) and treatment with RQ1 RNase-Free DNase I (Promega Corporation, Madison, WI, USA) according to the manufacturer’s instructions. Then, 0.5 μg of total RNA extracted from each sample was retrotranscribed to single strand cDNA by using the SensiFAST™ cDNA Synthesis Kit (Bioline Meridian Life Science, Memphis, TN, USA). Real-time reactions were performed in a total volume of 20 μl, including 1 μg of cDNA template, 400 nM of appropriate primer (Table 1), and 10 μl of iTaqTM Universal SYBR Green Supermix (BioRad Laboratories, Hercules, CA, USA). PCR cycling conditions were performed in a LightCycler 480 (Roche Diagnostics International, Rotkreuz, Switzerland) as follows: a cycle of 95°C for 30 s, then 45 cycles of 95°C for 5 s, and 60°C for 30 s, followed by a ramp from 60 to 95°C for melting curve stage. For each gene target, *n* ≥ 3 qPCR reactions were conducted, each reaction in duplicate.

**Table 1:** Primers used for RT-qPCR.

Gene	PCR primers	Sequence (5′-3′)	PCR product size (bp)
16S	chr16S-F	TACTACAATGCTACGGACAA	83
	chr16S-R	CCTGCAATCTGAACTGAG	
*uvrA*	chruvrA-F	ACTTAGATGTGATTCGTTGT	102
	chruvrA-R	CTACTTGCTCTGGTGTTC	
*uvrB*	chruvrB-F	CGATTACTATCAACCAGAAG	91
	chruvrB-R	CCGTAGCATATCAATCTCA	
*uvrC*	chruvrC-F	ACGGATACAGAAGCAGAA	81
	chruvrC-R	CTTGAGCAGCACATTGAA	
*uvsE*	chruvsE-F	TGTCCTTAGTTCTGATTCG	90
	chruvsE-R	GGTAAGCCTAACAAGTCA	
*phrA*	chrphrA-F	TTGGAGTAATTGGCATTCG	83

Relative mRNA levels were calculated by the comparative Ct method. Primer specificity was confirmed by melting curve analysis. 16S rRNA (GenBank accession number AF279107) was used as reference gene (Pinto et al., 2012). For dried-rewetted biofilms, levels of gene expression of DNA repair genes were measured after 30 and 60 min of rehydration, while 0-min recovery control was obtained from dried biofilms incubated in ice and resuspended in 1 ml of TRI Reagent (Sigma Aldrich, Saint Louis, MO, USA), as reported above. Values obtained for dried biofilms at 0-min recovery were set as 1. For dried-UV-irradiated-rewetted biofilms, levels of gene expression of DNA repair genes were measured after 30 min and 60 of rehydration, and the corresponding values of dried-rewetted biofilms were set as 1. Values were considered to be up-regulated (>1) or down-regulated (<1).

## Results

### Survivors Among Dried Biofilms and Dried-UV-Irradiated Biofilms

**Figure 2:**
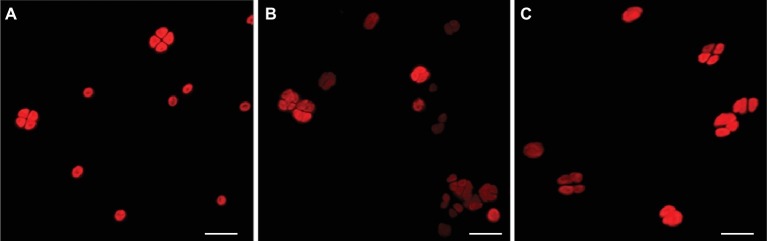
CLSM images of photosynthetic pigment autofluorescence. Cells from liquid cultures **(A)**; dried biofilms **(B)**; and dried-UV-irradiated biofilms **(C)**. Scale bar: 10 μm.

After 7 years of air-dried storage, the morphology of dried biofilms and dried-UV-irradiated biofilms (exposed to 1.5 × 10^3^ kJ/m^2^ of a Mars-like UV flux) was evaluated at the CLSM and compared to that of cells form liquid cultures used as control (Figure 2A). In dried biofilms (Figure 2B) as well as in UV-irradiated biofilms (Figure 2C), cells with an intense photosynthetic pigment autofluorescence (due to chlorophyll *a* and phycobiliproteins) occurred among bleached cells.

**Figure 3:**
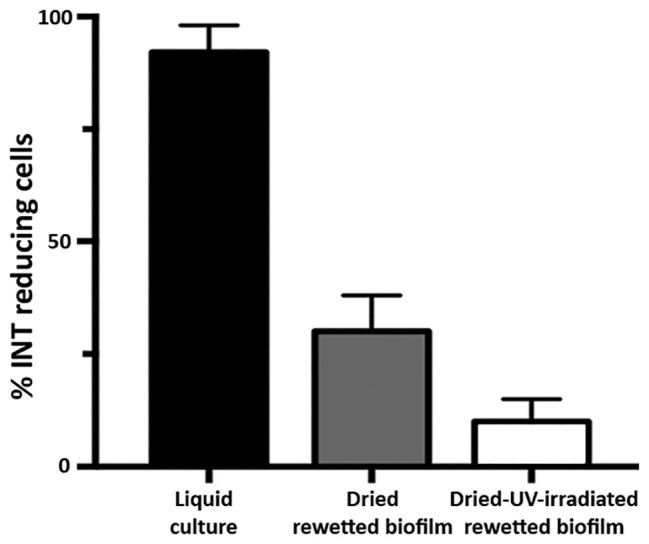
Alive cells in dried biofilms and dried-UV-irradiated biofilms as revealed by INT reduction after 72 h of rewetting.

Dried-rewetted biofilms and dried-UV-irradiated-rewetted biofilms were tested for respiration by monitoring the INT reduction by dehydrogenases after 72 h of rehydration. The INT staining revealed 30 and 10% of alive cells with insoluble red formazan spots in the cytoplasm of dried-rewetted biofilms and dried-UV-irradiated-rewetted biofilms, respectively, (Figure 3). On the contrary, INT reduction was undetectable after 30 and 60 min of rehydration (not shown).

### Increased DNA Damage in Dried-UV-Irradiated Biofilms Compared to Dried Biofilms

The presence of DNA damage in dried biofilms and in dried-UV-irradiated biofilms (exposed to 1.5 × 10^3^ kJ/m^2^ of a Mars-like UV flux) was qualitatively evaluated after 7 years of air-dried storage, by testing the genomic DNA suitability as template in PCR amplifications of short and long targets.

**Figure 4:**
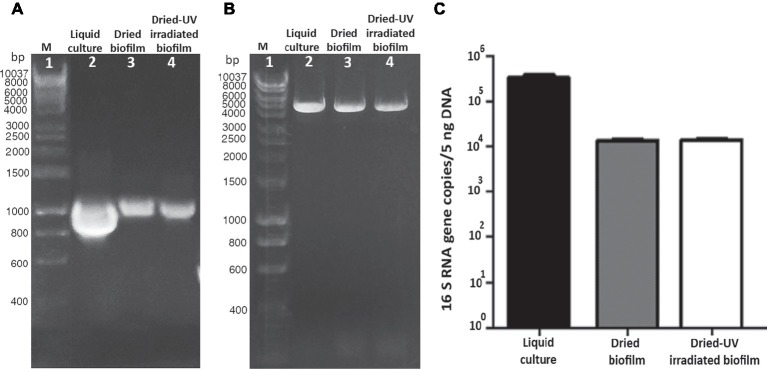
Assessment of DNA damage in cells from liquid culture, dried-biofilm, and dried-UV-irradiated biofilm. PCR amplification of 1,027-bp fragment of the16S rRNA gene **(A)** and 4-kbp genomic fragment **(B)**; lane 2: control cells from liquid culture; lane 3: dried biofilm; lane 4: dried-UV-irradiated biofilm; lane 1: Hyperladder 1 kbp (Bioline Meridian Life Science, Memphis, TN, USA). qPCR by using as target gene a 1,027-bp fragment of the 16S rRNA gene **(C)**.

In dried biofilms, the 1,027-bp amplification yielded a PCR amplicon of reduced intensity (Figure 4A, lane 3) compared to cells from liquid cultures (Figure 4A, lane 2). In dried-UV-irradiated biofilms, no additional decrease in the band intensity was detected with 1,027-bp amplification (Figure 4A, lane 4).

The amplification of a 4-kbp fragment yielded an amplicon of reduced intensity in dried biofilms (Figure 4B, lane 3) compared to cells form liquid cultures (Figure 4B, lane 2). While the intensity of the 4-kbp amplicon from dried-UV-irradiated biofilms was slightly reduced compared to dried biofilms (Figure 4B, lane 4).

When genomic DNA damage was quantified by means of qPCR, by using the 1,027-bp fragment as target, a significant reduction in the amplified copy number occurred in dried biofilms compared to cells form liquid culture, while no additional reduction was detected in dried-UV-irradiated biofilms (Figure 4C).

### Identification of UV-Damage DNA Repair Genes

The *in silico* analysis of *Chroococcidiopsis* genome identified sequences homologous to genes involved in three repair pathways of UV-induced DNA damage, namely photoreactivation, nucleotide excision repair, and UV damage endonuclease (UvsE)-dependent excision repair (Table 2).

**Table 2:** UV-induced DNA damage repair genes of *Chroococcidiopsis* sp. CCMEE 029 investigated in this study.

Gene name	Protein function	Gene length (nt)	Genbank accession number
*phrA*	deoxyribodipyrimidine photolyase	1,434	MK135046
*uvsE*	UV-damage endonuclease	981	MK135047
*uvrA*	UvrA, excinuclease ABC subunit A	3,033	MK135048
*uvrB*	UvrB, excinuclease ABC subunit B	2,004	MK135049
*uvrC*	UvrC, excinuclease ABC subunit C	1,923	MK135050

The *phrA* gene has a length of 1,434 bp with the highest similarity (BlastN output: query cover 89%, e-value 0.0, total score 791, and identity 78%) to the homolog in *Scytonema* sp. HK-05 (Genbank accession number AP018194.1; 7105541-7106989), encoding a deoxyribodipyrimidine photolyase.

The *uvsE* gene with a length of 981 bp showed the highest similarity (BlastN output: query cover 87%, e-value 2e-84, total score 326, and identity 74%) to the UV endonuclease *uvdE* gene of *Cylindrospermum* sp. NIES-4074 (Genbank accession number AP018269.1), encoding a UV damage endonuclease.

The *uvrA*, *uvrB*, and *uvrC* genes of *Chroococcidiopsis* have a length of 3,033; 2,004; and 1,923 bp, respectively. The *uvrA* gene shared the highest similarity (BlastN output: query cover 52%, e-value 0.0, total score 933 and identity 77%) with the homolog in *Nostoc* sp. PCC 7524 (Genbank accession number CP003552.1), encoding the excinuclease ABC subunit A. The *uvrB* gene showed the highest similarity (BlastN output: query cover 98%, e-value 0.0, total score 1,369 and identity 79%) to the homolog in *Nostoc commune* HK-02 (Genbank accession number AP018326.1; 7094877-7096874), encoding the excinuclease ABC subunit B. The *uvrC* gene shared the highest similarity (BlastN output: query cover 96%, e-value 0.0, total score 1,062 and identity 77%) to the homolog in *Fremyella diplosiphon* NIES-3275 (Genbank accession number AP018233.1; 1447846-1449714), encoding the excinuclease ABC subunit C.

### Different Expression of UV-Damage DNA Repair Genes in Dried-Rewetted Biofilms and Dried-UV-Irradiated-Rewetted Biofilms

**Figure 5:**
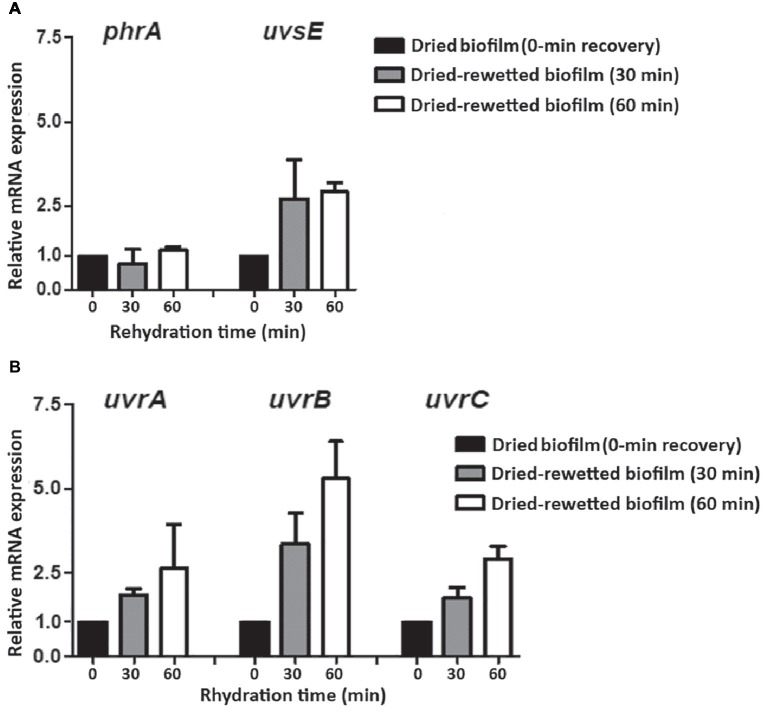
Expression of DNA repair genes in dried-rewetted biofilm after 30 and 60 min of rehydration. Expression of the *phrA* and *uvsE* genes **(A)** and of *uvrA, uvrB*, and *uvrC* genes **(B)**. Values from dried biofilms (0-min rewetting) were considered as control values set to 1. Subsequent samples were compared in terms of fold regulation to control values.

In order to evaluate the expression of the investigated DNA repair genes in the recovery of dried-rewetted biofilms, transcript levels detected in dried biofilms (0-min recovery) were set as 1 (Figure 5). The *uvsE* gene was up-regulated by 2.71- and 2.91-fold after 30 and 60 min of rewetting, whereas the *phrA* gene was not up-regulated (Figure 5A). The *uvrA*, *uvrB*, and *uvrC* genes were up-regulated: after 30 min of rewetting, the *uvrA* and *uvrC* genes were up-regulated by 1.84- and 1.74-fold, respectively, while the *uvrB* gene was up-regulated by 2.56-fold. After 60 min of rewetting, the *uvrA*, *uvrB*, and *uvrC* genes were up-regulated by 2.63-, 5.30-, and 2.91-fold, respectively, compared to dried biofilms (0-min recovery) (Figure 5B).

**Figure 6:**
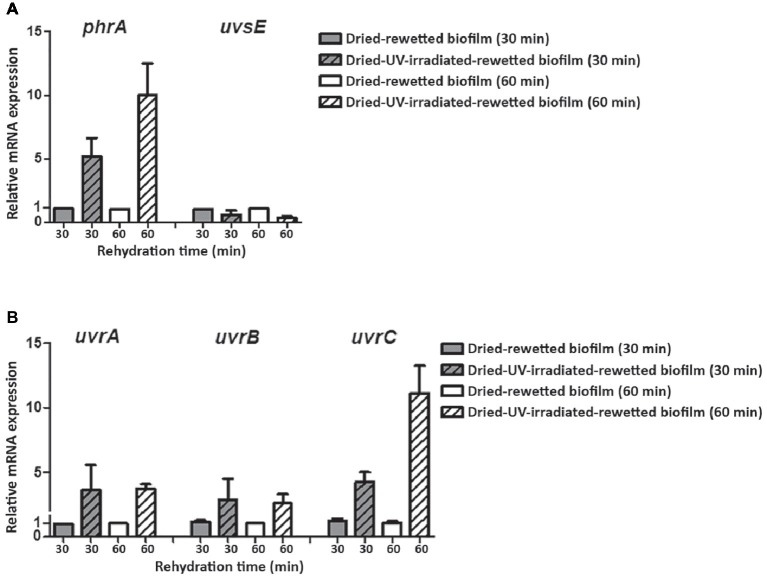
Expression of DNA repair genes in dried-UV-irradiated-rewetted biofilm after 30 and 60 min of rehydration. Expression of *phrA* and *uvsE* gene **(A)** and of *uvrA, uvrB*, and *uvrC* genes **(B)**. Values of dried-rewetted biofilms after 30 and 60 min of rehydration were considered as control values and set to 1. Subsequent samples were compared in terms of fold regulation to control values.

In order to evaluate the expression of the investigated DNA repair genes in the recovery of dried-UV-irradiated-rewetted biofilms, transcript levels detected in dried-rewetted biofilms, rehydrated for the same period of time, were set as 1 (Figure 6). The *uvsE* gene was not up-regulated in dried-UV-irradiated-rewetted biofilms; whereas the *phrA* gene was up-regulated by 5.19- and 9.98-fold after 30 and 60 min of rewetting, respectively (Figure 6A). Nucleotide excision repair genes were up-regulated after 30 and 60 min of rewetting, with the highest expression of the *uvrC* gene compared to *uvrA* and *uvrB* genes. In particular, after 30 min of recovery, the *uvrA* and *uvrB* genes were up-regulated by 3.61- and 2.85-fold, respectively; these expression levels remained almost the same after 60 min of recovery (3.72- and 2.59-fold, respectively). Whereas, the *uvrC* gene was over-expressed by 4.26- and 11.12-fold after 30 and 60 min of recovery compared to dried-rewetted biofilms, at the same recovery points (Figure 6B).

## Discussion

Here, the desiccation and UV tolerance limits of *Chroococcidiopsis* sp. CCMEE 029 were stretched further by the recovery capability of dried biofilms and dried-UV-irradiated biofilms (exposed to 1.5 × 10^3^ kJ/m^2^ of a Mars-like UV flux) after 7 years of air-dried storage. This astonishing performance extends our knowledge of biofilm endurance, already recognized as the most successful life forms on Earth (Flemming and Wingender, 2010).

In order to unravel the mechanisms underlying such endurance, the role of both protection mechanisms taking place upon desiccation and repair mechanisms triggered upon rehydration must be taken into consideration. In fact, although *Chroococcidiopsis* adopts efficient countermeasures to avoid the otherwise lethal effects of water removal, its endurance when air-dried can be limited by the oxidative damage accumulated even in the absence of metabolic activity (Billi, 2009). In *Chroococcidiopsis* sp. CCMEE 029, the avoidance of protein oxidative damage was identified as a first line of defense against desiccation and ionizing radiation (Fagliarone et al., 2017). Indeed the degree of bacterial resistance to desiccation and radiation depends on the level of oxidative damage to proteins, including those needed to repair extensive DNA damage that prevents transcription and translation (Slade and Radman, 2011). In addition, during UV irradiation, dried cells accumulated DNA lesions, such us cyclobutane pyrimidine dimers and pyrimidine-pyrimidone (6–4) photoproducts and 8-oxo-7, 8-dihydroguanine cyclobutane pyrimidine (Goosen and Moolenaar, 2008), all of which must be repaired upon rehydration.

In the present work, the presence of DNA damage in dried biofilms and dried-UV-irradiated biofilms was evaluated after 7 years of desiccation, by testing the genome suitability as template in PCR-stop assays by using short and long PCR targets. The principle is that DNA damage inhibits PCR by impairing DNA polymerase progression (Kumar et al., 2004), while PCR targets of different lengths affect the likelihood of encountering a DNA damage, short amplicons having a lower likelihood than long amplicons (Rudi et al., 2010). The presence of DNA lesions in dried biofilms compared to cells from liquid cultures was revealed by a reduced intensity of the 1,027-bp and 4-kbp PCR band. No further reduction of the 1,027-bp PCR band intensity was detected in dried-UV-irradiated biofilms when using PCR-stop assay with the 1,027-bp target. Also, qPCR using the 1,027-bp target did not reveal any increase of the DNA damage in dried-UV-irradiated biofilms compared to dried biofilms. However, the reduced intensity of the 4,000-bp PCR band suggested an increased accumulation of DNA lesions in dried-UV-irradiated biofilms compared to dried biofilms. Anyway, the possibility to perform PCR amplifications is in agreement with the lack of genome degradation, previously reported for this cyanobacterium after 4 years of air-drying (Billi, 2009).

After 7 years of air-drying, *Chroococcidiopsis* not only avoided genome degradation but preserved at least a sub-set of mRNAs and 16S ribosomal RNA. This persistence is relevant if compared to that of desiccation-tolerant cyanobacteria dried for shorter periods. The absence of RNA fragmentation was reported for the desert cyanobacterium *Gloeocapsopsis* AAB1 desiccated for 13 days (Azua-Bustos et al., 2014), and the stable maintenance of mRNAs through dormancy was reported for Microcoleus vaginatus (Rajeev et al., 2013). Detectable ribosomal RNA and mRNAs, including abundant *sodF* mRNA, occurred in the cyanobacterium Nostoc commune dried for 3 years (Shirkey et al., 2000), while *in vitro* translation failed when using mRNA of Nostoc commune dried for 5 years (Jäger and Potts, 1988). Remarkably, a desiccation-sensitive cyanobacterium such as *Synechocystis* sp. PCC 6803 could not survive 3 months of air-dried storage (Fagliarone et al., 2017).

The presence of ribosome machinery is considered an indicator of cell viability and of a potential capability of a rapid response in a new environment conditions (Emerson et al., 2017). Alive 16S rRNA-containing cells were detected in dried biofilms of *Deinococcus geothermalis* (Frösler et al., 2017). Nucleic acid accumulation has been considered a requirement for cyanobacterial dormancy and germination (Kaplan-Levy et al., 2010). For example, *Aphanizomenon ovalisporum* akinetes showed a 10-fold increase in the volumetric ribosome content compared to vegetative cells (Sukenik et al., 2012).

In the present work, the occurrence of survivors in dried biofilms and dried-UV-irradiated biofilms was proved by growth after transfer into liquid BG-11 medium (not shown) and by INT reduction after 72 h of rewetting. These cells showed an intense autofluorescence of the photosynthetic pigments that were unable of INT reduction. Indeed after long-term (years of) desiccation, *Chroococcidiopsis* survivors were scored among dead cells that had bleached photosynthetic pigments, fragmented DNA, and degenerated ultrastructural features (Grilli Caiola, et al., 1993; Billi, 2009).

In the present work, a single-cell evaluation of the RNA content was not performed; nevertheless, the persistence of intact ribosome machinery and mRNAs might have contributed to *Chroococcidiopsis* biofilms’ resuscitation from prolonged dormancy, when respiration should have already started (Scherer et al., 1984; Higo et al., 2007). Further investigation into the presence of a “dormant transcriptome” in dried *Chroococcidiopsis* should be carried out under mRNA *de novo* synthesis arrest. A synergic role might have been played by the presence in dried *Chroococcidiopsis* cells of a proteome, including DNA repair proteins, that was protected against oxidative damage (Fagliarone et al., 2017). Indeed during the first hour of *Deinococcus radiodurans’*s recovery from ionizing radiation and desiccation, most DNA repair genes were not over-expressed, possibly due to a constitutive expression, sufficient to repair DNA damage, or due to proteins of unknown function (Tanaka et al., 2004).

In *Chroococcidiopsis*, the different up-regulation of the investigated DNA repair genes during the early phase recovery of dried biofilms and dried-UV-irradiated biofilms highlighted the accumulation of different types and/or amounts of DNA lesions. It also suggested that genes involved in the repair of UV-induced damage played a key role in the recovery from desiccation.

The *phrA* gene was markedly over-expressed during the recovery of dried-UV-irradiated biofilms, supporting the relevance of the codified photolyase in repairing cyclobutane pyrimidine dimers. By contrast, the *uvsE* gene, encoding a putative UV damage endonuclease, showed the highest over-expression during the recovery of dried biofilms. The UvsE-dependent excision repair is not common in cyanobacteria (Goosen and Moolenaar, 2008; Cassier-Chauvat et al., 2016), although its role in repairing UV-induced DNA damage was reported for *Deinococcus radiodurans* (Tanaka et al., 2005). However, since UvsE recognizes also non-UV-induced DNA damage such as abasic sites, nicks, and gaps (Meulenbroek et al., 2013), it might be involved in *Chroococcidiopsis* in repairing desiccation-induced DNA damage rather than UV-induced damage, thus reflecting a redundancy in order to counteract desiccation-induced DNA damage.

Dried-rewetted biofilms showed the up-regulation of the nucleotide excision repair genes encoding UvrA and UvrB, both involved in damage recognition and UvrC for the incision on either side of the lesions (Goosen and Moolenaar, 2008). Also, dried-UV-irradiated rewetted biofilms showed an increased expression of these genes, *uvrC* showing the highest value. A higher *uvrC* gene expression was reported for *Halococcus hamelinensis* during the first hour of UV-induced damage repair and it was suggested that *uvrA* and *uvrB* genes were constitutively expressed due to their having other roles in addition to that of DNA repair (Leuko et al., 2011). The role of the nucleotide excision repair in desiccation tolerance was highlighted in *Sinorhizobium meliloti* in which the inactivation of the *uvrA*, *uvrB*, and *uvrC* genes resulted in desiccation-sensitive mutants (Humann et al., 2009). Moreover, during the first hour of *Deinococcus radiodurans’*s recovery from ionizing radiation and desiccation *uvrA*, and *uvrB* genes were included in the 32 foci over-expressed (Tanaka et al., 2004).

The high similarity between the investigated DNA repair genes of *Chroococcidiopsis* sp. CCMEE 029 and homologs in filamentous, heterocystous cyanobacteria is in agreement with the phylogenetic analysis reporting that the unicellular non-heterocyst-differentiating genus *Chroococcidiopsis* and the filamentous heterocyst-differentiating cyanobacteria are each other’s closest living relatives (Fewer et al., 2002). Moreover, among the strains with the highest sequence similarities occurred isolates from extreme environments such as *Scytonema* sp. HK-05 (genebank synonym *Scytonema* sp. NIES-2130) from a hot spring (Rippka et al., 1979) and *Nostoc* sp. HK-01 from natural cyanobacterial crusts (Katoh et al., 2012).

In the present work, the genome sequencing of *Chroococcidiopsis* sp. CCMEE 029 was undertaken; when the bioinformatics analysis will be completed and the genome resealed, key signatures for its desiccation and radiation tolerance will be identified, as recently reported for *Gloeocapsopsis* sp. UTEX B3054 (Urrejola et al., 2019). Preliminary bioinformatics analysis (not shown) pointed out that unlike other cyanobacteria (Cassier-Chauvat et al., 2016) and similar to *Deinococcus radiodurans* (Timmins and Moe, 2016), the genome of *Chroococcidiopsis* sp. CCMEE 029 lacks *recB* and *recC* genes that are involved in the homologous recombination (Spies and Kowalczykowski, 2005). Hence further investigations are needed to unravel additional pathways involved in the repair of DNA damage induced by UV irradiation and desiccation.

Reshaping the boundaries of *Chroococcidiopsis* desiccation and UV tolerance has implications in the search for extra-terrestrial life since it contributes to defining the habitability of Mars and planets orbiting other stars. In fact, the UV dose used here corresponds to that of a few hours at Mars’s equator (Cockell et al., 2000). Hence, considering that survivors occurred in the bottom layers of the biofilms (Baqué et al., 2013), it might be hypothesized that if a biofilm life form ever appeared during Mars’s climatic history, it might have been transported in a dried state under UV radiation, from niches that had become unfavorable to niches that were inhabitable (Westall et al., 2013). The reported survival also suggests that intense UV radiation fluxes would not prevent the presence of phototrophic biofilms or their colonizing of the landmass of other planets.

## Data Availability Statement

The raw data supporting the conclusions of this manuscript will be made available by the authors, without undue reservation, to any qualified researcher.

## Author Contributions

DB and LR supervised the study. CM performed the experiments, CF and MB contributed to the materials and analysis tool. ER and PR conceived and performed the martian UV radiation simulation. AN, FF and MP carried out the bioinformatic analyses. DB wrote the manuscript. All authors read and approved the final manuscript.

### Conflict of Interest

The authors declare that the research was conducted in the absence of any commercial or financial relationships that could be construed as a potential conflict of interest.

## Footnotes

^1^https://orione.crs4.it
